# Requirement for ER-mitochondria Ca^2+^ transfer, ROS production and mPTP formation in L-asparaginase-induced apoptosis of acute lymphoblastic leukemia cells

**DOI:** 10.3389/fcell.2023.1124164

**Published:** 2023-02-21

**Authors:** Jung Kwon Lee, Jesusa L. Rosales, Ki-Young Lee

**Affiliations:** Department of Cell Biology and Anatomy, Arnie Charbonneau Cancer and Alberta Children’s Hospital Research Institutes, University of Calgary, Calgary, AB, Canada

**Keywords:** blood-related disorders, leukemia, acute lymphoblastic leukemia, chemotherapy, L-asparaginase

## Abstract

Acute lymphoblastic leukemia (aLL) is a malignant cancer in the blood and bone marrow characterized by rapid expansion of lymphoblasts. It is a common pediatric cancer and the principal basis of cancer death in children. Previously, we reported that L-asparaginase, a key component of acute lymphoblastic leukemia chemotherapy, causes IP3R-mediated ER Ca^2+^ release, which contributes to a fatal rise in [Ca^2+^]_cyt_, eliciting aLL cell apoptosis *via* upregulation of the Ca^2+^-regulated caspase pathway (Blood, 133, 2222–2232). However, the cellular events leading to the rise in [Ca^2+^]_cyt_ following L-asparaginase-induced ER Ca^2+^ release remain obscure. Here, we show that in acute lymphoblastic leukemia cells, L-asparaginase causes mitochondrial permeability transition pore (mPTP) formation that is dependent on IP3R-mediated ER Ca^2+^ release. This is substantiated by the lack of L-asparaginase-induced ER Ca^2+^ release and loss of mitochondrial permeability transition pore formation in cells depleted of HAP1, a key component of the functional IP3R/HAP1/Htt ER Ca^2+^ channel. L-asparaginase induces ER Ca^2+^ transfer into mitochondria, which evokes an increase in reactive oxygen species (ROS) level. L-asparaginase-induced rise in mitochondrial Ca^2+^ and reactive oxygen species production cause mitochondrial permeability transition pore formation that then leads to an increase in [Ca^2+^]_cyt_. Such rise in [Ca^2+^]_cyt_ is inhibited by Ruthenium red (RuR), an inhibitor of the mitochondrial calcium uniporter (MCU) that is required for mitochondrial Ca^2+^ uptake, and cyclosporine A (CsA), an mitochondrial permeability transition pore inhibitor. Blocking ER-mitochondria Ca^2+^ transfer, mitochondrial ROS production, and/or mitochondrial permeability transition pore formation inhibit L-asparaginase-induced apoptosis. Taken together, these findings fill in the gaps in our understanding of the Ca^2+^-mediated mechanisms behind L-asparaginase-induced apoptosis in acute lymphoblastic leukemia cells.

## Introduction

Acute lymphoblastic leukemia (aLL) is a devastating cancer of immature lymphocytes. It largely afflicts children, representing more than a quarter of all childhood cancers, and causing most of the fatalities from cancer in children (Hunger and Mullighan, 2015). L-asparaginase is a key component of aLL chemotherapy. Regimens that consist of L-asparaginase give rise to greater induction of remission compared to L-asparaginase-free regimens (Egler et al., 2016). L-asparaginase is thought to trigger asparagine insufficiency, causing protein synthesis inhibition and subsequent aLL cell death. However, treatment of L-asparaginase comes with the risk of resistance.

Using genome-wide RNA interference screening, we discovered huntingtin-associated protein 1 (HAP1) (Lee et al., 2019) as a novel biomarker for L-asparaginase resistance in aLL cells. Loss of HAP1 expression in aLL patient primary leukemic cells corresponds to L-asparaginase resistance, indicating that L-asparaginase induces aLL cell apoptosis (Kang et al., 2017; Lee et al., 2019) through a novel non-canonical pathway that involves HAP1. HAP1 binds to huntingtin (Htt) and the intracellular inositol 1,4,5- trisphosphate (IP3) receptor (IP3R) Ca^2+^ channel to form a functional HAP1-Htt-IP3R complex that regulates IP3-stimulated ER Ca^2+^ release. HAP1 loss inhibits HAP1-Htt-IP3R formation and thus L-asparaginase stimulation of ER Ca^2+^ release (Lee et al., 2019). Loss of HAP1 also reduces entry of external Ca^2+^, inhibiting an overwhelming increase in [Ca^2+^]_i_, and downregulating the Ca^2+^-activated calpain 1, Bid, and caspase-3/12 apoptotic pathway, which result in L-asparaginase resistance (Lee et al., 2019). These findings indicate that L-asparaginase causes aLL cell apoptosis through perturbation of intracellular Ca^2+^ homeostasis and subsequent upregulation of the Ca^2+^-activated calpain 1, Bid, and caspase-3/12 apoptotic pathway. The ability of the Ca^2+^ chelator, BAPTA-AM, to almost completely reverse aLL cell apoptosis establishes an association between an increase in [Ca^2+^]_cyt_ and L-asparaginase-stimulated apoptosis (Lee et al., 2019). However, the cellular events that lead to a lethal increase in [Ca^2+^]_i_ following L-asparaginase-stimulated ER Ca^2+^ release are still unknown.

Mitochondria are cell organelles that regulate Ca^2+^ homeostasis and apoptosis. The outer mitochondrial membrane (OMM) is easily permeable to Ca^2+^ while the inner mitochondrial membrane (IMM) consists of the mitochondrial calcium uniporter (MCU) complex that mediates mitochondrial Ca^2+^ influx (Collins et al., 2001). As MCU has low affinity to Ca^2+^, increased Ca^2+^ concentration is required for MCU activity (Paupe and Prudent, 2018). Uptake of mitochondrial Ca^2+^ *via* MCU channels is made possible by the proximity between the mitochondria and the ER (Rizzuto et al., 2009; Grimm, 2012), the major intracellular Ca^2+^ store. A typical mechanism for ER-mitochondria communication is *via* the mitochondria-associated ER membrane (MAM) (Vance, 2014), the ER-mitochondria interface. MAMs are associated with several proteins such as IP3R Ca^2+^ channels (Patergnani et al., 2011) and voltage-dependent anion channels (VDACs) (Ma et al., 2017). These channels regulate ER Ca^2+^ transport to the mitochondria (Patergnani et al., 2011). Once ER Ca^2+^ is released through IP3R channels, mitochondria Ca^2+^ uptake occurs (Patergnani et al., 2011) *via* the OMM VDACs, and the IMM MCU channel (Rizzuto et al., 2009; Shoshan-Barmatz et al., 2017). However, overload of mitochondrial Ca^2+^ is related to not only increased or sustained formation of the mitochondrial permeability transition pore (mPTP) (Moore, 1971; Duchen, 2000; Finkel et al., 2015) but also the generation of mitochondrial ROS (Ermak and Davies, 2002; Gorlach et al., 2015), which also contributes to mPTP formation (Zorov et al., 2000; NavaneethaKrishnan et al., 2020) that allows ROS release into the cytoplasm (Zorov et al., 2014). The mPTP channel regulates the IMM permeabilization. Although transient mPTP opening serves as a mitochondrial Ca^2+^ efflux channel under normal conditions (Altschuld et al., 1992; Ichas et al., 1997), sustained mPTP formation triggers swelling of mitochondria and secretion of cytochrome C and other intermembrane space (IMS) proteins, causing caspase-regulated apoptosis (Lemasters et al., 2002; Kinnally et al., 2011).

In the current study, we utilized SEM patient aLL cells expressing or depleted of HAP1 by retroviral transfection, and demonstrate that L-asparaginase-induced aLL cell apoptosis triggered by a lethal rise in [Ca^2+^]_cyt_ is caused by mPTP formation that results from ER-mitochondria Ca^2+^ transfer and subsequent ROS production. Thus, our findings define the Ca^2+^-mediated mechanisms through which L-asparaginase perturbs intracellular Ca^2+^ homeostasis to cause apoptosis in aLL cells.

## Materials and methods

### Materials

RPMI 1640 media, fetal bovine serum, penicillin-streptomycin, Mag-Fluo-4 AM, Rhod-2 AM, Fluo-4 AM, Annexin V-FITC staining kit, Image-IT live mitochondria permeability transition pore assay kit, MitoSOX Red, MitoTracker green and DCFDA were from Thermo Fisher Scientific (Burlington, ON, Canada). L-asparaginase (ab73439) was from Abcam (Toronto, ON, Canada). 2,5-di-tert-butylhydroquinone (TBHQ) was from Sigma (Oakville, ON, Canada). Xestospongin-C (XeC), ruthenium red (RuR), and cyclosporine A (CsA) were from Bio-Techne (Oakville, ON, Canada). HAP1 (D-12) and actin (I19) antibodies, and Mito-Tempo were from Santa Cruz Biotech. (Dallas, TX, United States of America).

### Cell culture

SEM cells were originally derived from a relapsed 5-year-old female patient diagnosed with pre-B aLL (Greil et al., 1994). These cells, which were prepared by high-density culture of blast cells, exhibited continuous growth and survival *in vitro* (Lee et al., 2019). SEM cells (*) infected with retrovirus carrying an empty pRS vector (*+pRS) or pRS-sh*HAP1* (*+pRS-sh*HAP1*) were generated as we described previously (Lee et al., 2019). These cells were cultured in RPMI 1640, containing 10% FBS and 100 μg/ml penicillin-streptomycin, at 37°C in 5% CO_2_.

### mPTP formation

Formation of mPTP was assessed using the Image-IT live mitochondria permeability transition pore assay kit following the manufacturer’s instructions. *+pRS or *+pRS-sh*HAP1* cells (0.1 × 10^6^) loaded with 1 µM calcein-AM then pre-treated with 3 μM RuR or 1 μM CsA for 10 min were stimulated with 100 mIU L-asparaginase for 30 min then treated with 1 mM CoCl_2_ for 15 min. Treatments were performed at 37°C. Cells were rinsed in HBSS, resuspended in ice-cold 1x PBS, and analyzed by flow cytometry using a fluorescein isothiocyanate filter (530 nm).

### Western blot analysis

Cell lysates were resolved by 12.5% SDS-PAGE, transferred to a nitrocellulose membrane, and immunoblotted using the indicated antibodies. Western blot images were captured using a ChemiDoc Imager (Bio-Rad) set at optimal exposure. Chemiluminescence intensity ratios of protein bands of interest vs. actin were determined after densitometry of blots using the National Institutes of Health ImageJ 1.61 software.

### Ca^2+^ measurement

To measure ER Ca^2+^ release, *+pRS and *+pRS-sh*HAP1* cells (0.5 × 10^6^) loaded with 2.5 μM Mag-Fluo-4 AM [in Ca^2+^-free Krebs-Ringer-Henseleit (KRH) buffer containing 25 mM HEPES, pH 7.4, 125 mM NaCl, 5 mM KCl, 6 mM glucose, and 1.2 mM MgCl_2_ + 5 μM EGTA] for 30 min then stimulated with 100 mIU L-asparaginase were analyzed using a Shimadzu RF 5301 PC spectrofluorometer (Tokyo, Japan) at λ_ex_ = 495_nm_ and λ_em_ = 530_nm_.

To measure [Ca^2+^]_mt_, *+pRS and *+pRS-sh*HAP1* (0.5 × 10^6^) loaded with 2 μM of Rhod-2 AM [in Ca^2+^-free KRH buffer containing 5 μM EGTA] for 1 h then pre-treated with 2 μM XeC or 3 μM RuR were stimulated with 100 mIU L-asparaginase and analyzed using a Shimadzu RF 5301 PC spectrofluorometer at λ_ex_ = 550_nm_ and λ_em_ = 588_nm_. Peak amplitudes were quantified as ratios of fluorescence (F/F_0_) after addition of L-asparaginase. F_0_ represents basal fluorescence or fluorescence before stimulation with L-asparaginase.

To measure [Ca^2+^]_cyt_,*+pRS and *+pRS-sh*HAP1* cells (0.5 × 10^6^) grown on poly-L-ornithine-coated glass coverslips were loaded with 5 μM Fluo-4 AM [in Ca^2+^-free KRH buffer] for 1 h then pre-treated with 2 μM XeC, 3 μM RuR or 1 μM CsA and stimulated with 100 mIU L-asparaginase. Ca^2+^ transients were analyzed by single-cell Ca^2+^ imaging using an Olympus X71 inverted microscope (Tokyo, Japan) at λ_ex_ = 485 nm and λ_em_ = 530 nm. Fluorescence intensities were measured in individual cells (n = 10) every 2 s. Data were analyzed using ImageJ 1.4.1 (NIH, United States of America). The integrated Ca^2+^ signals (area under the curve0 were calculated at 60 s–240 s following treatment.

### Measurement of Reactive Oxygen Species (ROS)

*+pRS and *+pRS-sh*HAP1* cells seeded on poly-ornithine coated coverslips and pre-treated with RuR (3 μM), CsA (1 μM) or Mito-Tempo (5 μM) then stimulated with 100 mIU L-asparaginase for 12 h were stained with MitoSOX red (5 μM) and MitoTracker green (200 nM) or DCFDA (5 μM) for 30 min at 37°C. MitoTracker green was used to label mitochondria in live cells. Cell images were acquired using an Olympus 1 × 71 inverted microscope (Tokyo, Japan) at 160 to ×360 magnification. Fluorescence intensity of captured images (from a field with at least 200 cells) were measured using the ImageJ software. Values from cells stimulated with L-asparaginase alone were normalized to 1.

### Apoptosis

*+pRS and *+pRS-sh*HAP1* cells (1×10^4^) seeded on 96-well plates coated with 0.2 mg/ml poly-L-ornithine and pre-treated with RuR (3 μM), CsA (1 μM) or Mito-Tempo (5 μM) for 30 min then stimulated with 100 mIU L-asparaginase for 12 h were stained with Hoechst 34580 and FITC-Annexin V. FITC-positive apoptotic cells were counted 12 h post-treatment at ×10 magnification using a I×71 Olympus inverted microscope attached to a 37°C incubator with 5% CO_2_. The percentage of FITC-positive apoptotic cells was determined from a field of ∼100 Hoechst 34580-stained cells using the Olympus CellSens software (Olympus, Japan).

### Statistical analysis

Student’s t-test (unpaired, two-tailed) was performed at *p* < 0.05 for experiments involving two treatment groups. For experiments involving more than two treatment groups, one-way Analysis of Variance (ANOVA) with Tukey Honestly Significantly Different (HSD) *post hoc* tests were performed.

## Results

**FIGURE 1:**
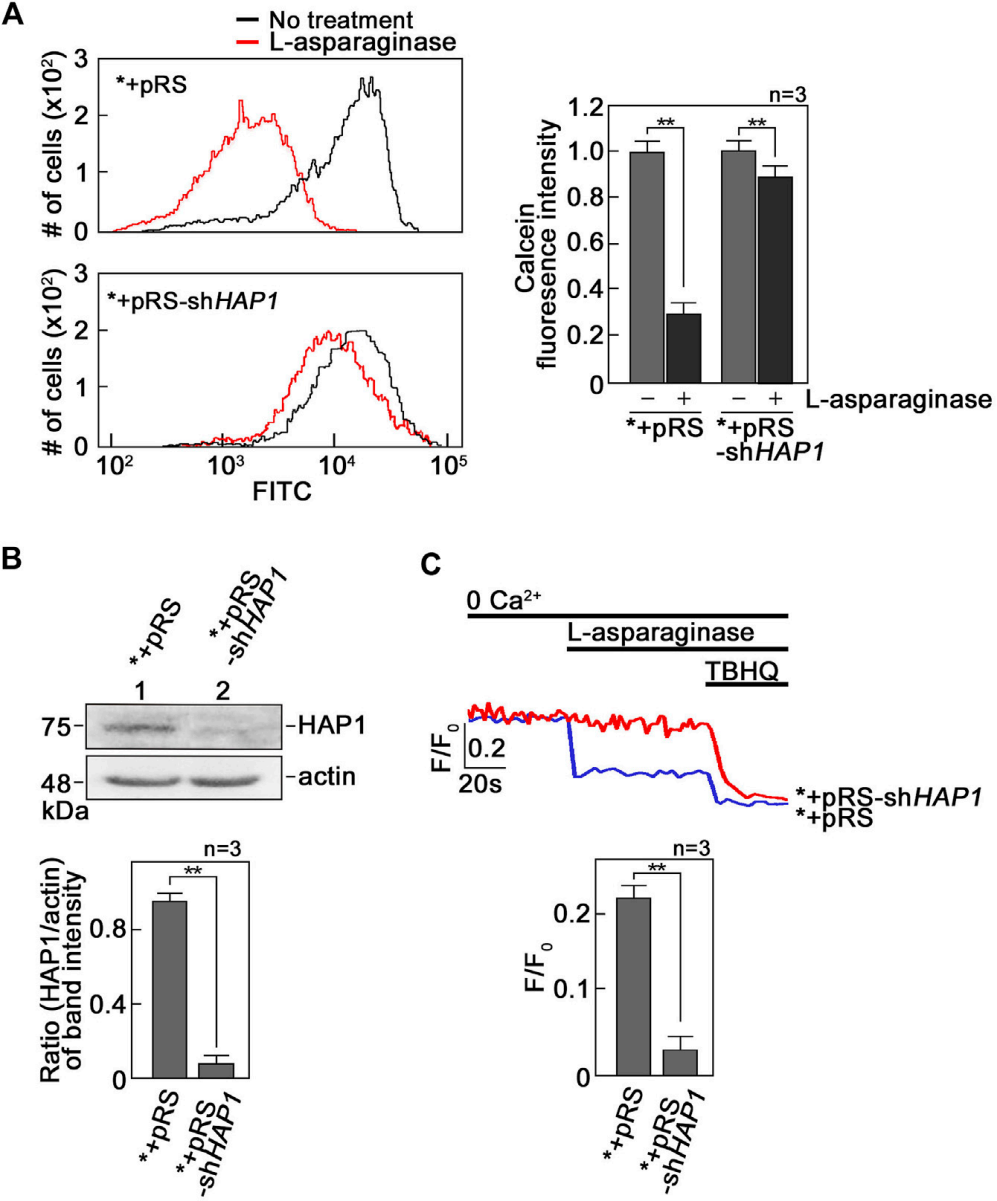
L-asparaginase-induced IP3R-mediated ER Ca^2+^ release causes mPTP formation. **(A)** SEM cells (*) infected with retrovirus carrying an empty pRS vector (*+pRS) or pRS-sh*HAP1* (*+pRS-sh*HAP1*) and loaded with calcein-AM were stimulated with L-asparaginase. Cells were then treated with CoCl_2_ and subjected to flow cytometry analysis. Data on the left are from one of three independent experiments showing similar results. The chart on the right shows quantitative analysis of the relative calcein fluorescence in *+pRS and *+pRS-sh*HAP1* cells treated (or untreated) with L-asparaginase. Readings from untreated cells were normalized to 1.0. Values are means ± SEM from the three independent experiments (n = 3). **p < 0.05. **(B)** Lysates of *+pRS and *+pRS-sh*HAP1* cells were resolved by SDS-PAGE and immunoblotted for HAP1. Blots (upper panel) shown represent one of three blots with similar results. The actin blot serves as loading control. The bottom panel shows ratios of HAP1 vs. actin levels based on densitometric analysis of blots from the three independent experiments (*n* = 3) using the NIH ImageJ 1.61 software. Actin values were normalized to 1.0. **p < 0.05. **(C)** *+pRS and *+pRS-sh*HAP1* cells (0.5 × 10^6^ cells) loaded with Mag-Fluo-4 AM, an ER Ca^2+^ probe (Takahashi et al., 1999), then treated with L-asparaginase were analyzed for ER Ca^2+^ release by spectrofluorometry. Tracings on the upper panel are from one of three independent experiments showing similar results. The chart (bottom panel) shows ER Ca^2+^ release in *+pRS and *+pRS-sh*HAP1* cells stimulated with L-asparaginase. Values are means ± SEM from the three independent experiments (*n* = 3). **p < 0.05.

L-asparaginase-induced ER Ca^2+^ release that is mediated by IP3R causes mPTP formation. To investigate if L-asparaginase-induced IP3R-mediated ER Ca^2+^ release (Lee et al., 2019) causes mPTP formation, SEM cells (*) infected with retrovirus carrying an empty pRS vector (*+pRS) were loaded with calcein-AM and stimulated with L-asparaginase. Cells were then treated with CoCl_2_ and analyzed by flow cytometry. Cell-permeable calcein-AM dye disperses and gets confined into subcellular organelles such as mitochondria (Petronilli et al., 1998). CoCl_2_ removes calcein staining in all subcellular compartments except the mitochondria, which are surrounded by a CoCl_2_-resistant inner mitochondrial membrane (IMM), when mPTP is closed (Petronilli et al., 1998). Thus, CoCl_2_ treatment permits detection of status of mPTP formation (Petronilli et al., 1998). As shown in Figure 1A, L-asparaginase caused an obvious shift in calcein-stained population of *+pRS cells, indicating clear removal of calcein staining and, therefore, mPTP formation in these cells. To establish a link between L-asparaginase-induced IP3R-mediated ER Ca^2+^ release and mPTP formation, SEM cells (*) stably depleted of HAP1 by infection with retrovirus carrying pRS-sh*HAP1* (*+pRS-sh*HAP1*) (Lee et al., 2019) were used. Lack of HAP1 (Figure 1B), a key component of the functional ER Ca^2+^channel, IP3R/HAP1/Htt ternary complex (Lee et al., 2019), inhibited L-asparaginase-induced ER Ca^2+^ release (Figure 1C). Treatment with TBHQ, an ER Ca^2+^ pump inhibitor, caused ER Ca^2+^ release in both *+pRS and *+pRS-sh*HAP1* cells, indicating viability of these cells during analysis. In *+pRS-sh*HAP1* cells where ER Ca^2+^ release was blocked due to HAP1 loss, L-asparaginase caused a modest shift in calcein-stained population (Figure 1A), indicating high and greater retention of calcein staining in these cells compared to *+pRS cells, and, therefore, closed mPTP. These findings indicate that L-asparaginase-induced IP3R-mediated ER Ca^2+^ release, which was observed in *+pRS cells, causes mPTP formation.

**FIGURE 2:**
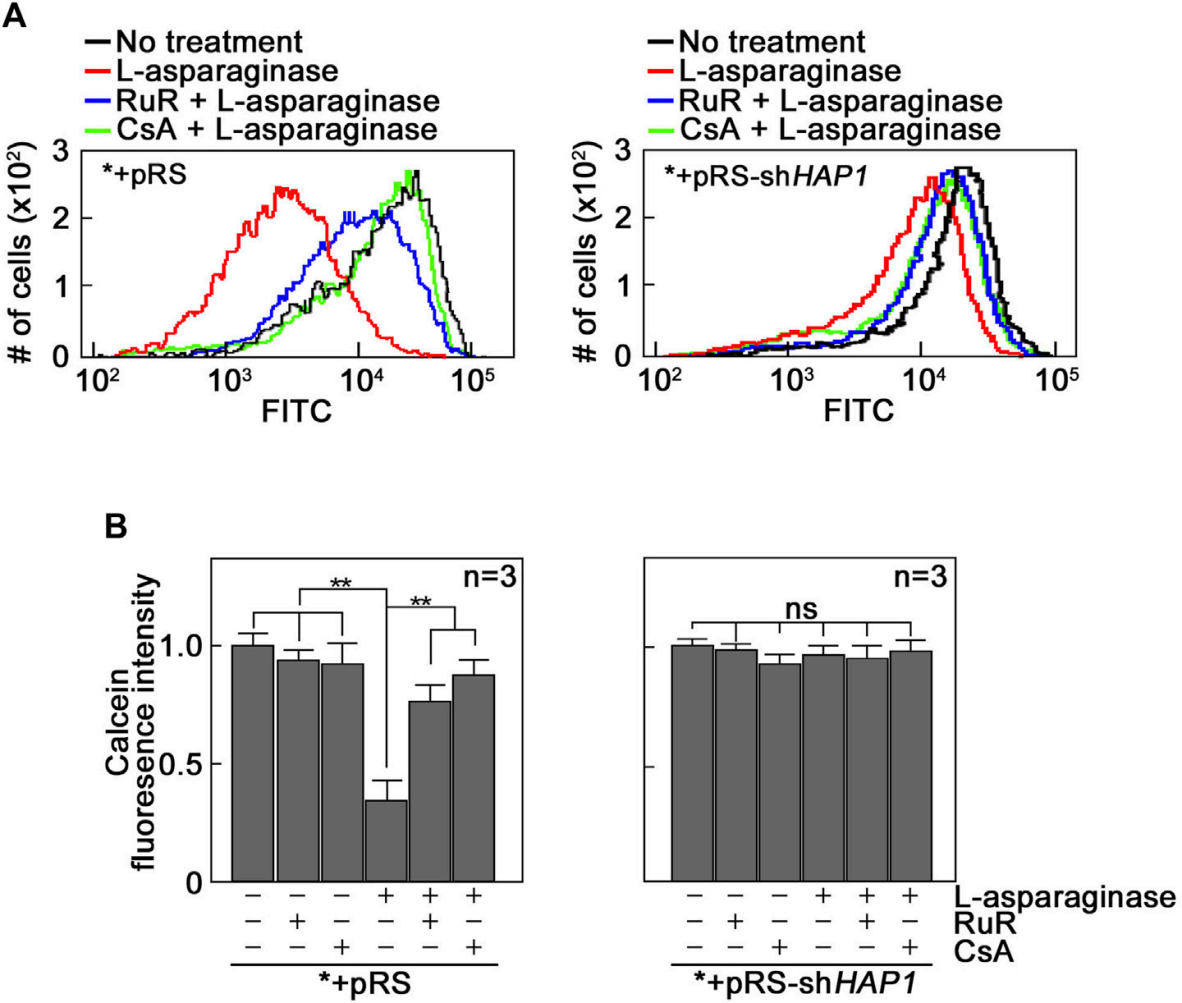
The MCU channel is involved in L-asparaginase-induced mPTP formation. *+pRS and *+pRS-sh*HAP1* cells loaded with calcein-AM then pre-treated with RuR or CsA were stimulated with L-asparaginase then treated with CoCl_2_. Quantitative analysis of the relative calcein fluorescence in *+pRS and *+pRS-sh*HAP1* cells was performed by flow cytometry. **(A)** Data are from one of three independent experiments showing similar results. **(B)** Readings from untreated cells were normalized to 1.0. Values are means ± SEM from the three independent experiments (*n* = 3). **p < 0.05.

L-asparaginase-induced mPTP formation, which is dependent on IP3R-mediated ER Ca^2+^ release, results from Ca^2+^ entry into mitochondria. Since mPTP formation is associated with Ca^2+^ overload in mitochondria (Duchen, 2000; Contreras et al., 2010; Finkel et al., 2015; NavaneethaKrishnan et al., 2020) that could be mediated by the MCU located in the IMM (Moore, 1971), we tested the involvement of MCU in L-asparaginase-induced mPTP formation. To do so, *+pRS and ^*^+pRS-sh*HAP1* cells pre-treated with Ruthenium Red (RuR), a potent MCU inhibitor (Moore, 1971), and stimulated with L-asparaginase were examined for mPTP formation as described above. As shown in Figures 2A, B, RuR increased calcein staining in *+pRS cells stimulated with L-asparaginase (left panel), indicating inhibition of L-asparaginase-induced mPTP formation. As expected, RuR had no effect on calcein fluorescence intensity in ^*^+pRS-sh*HAP1* cells stimulated with L-asparaginase (right panel). Cyclosporine A (CsA), an mPTP inhibitor (Crompton et al., 1988), was used as positive control. These findings indicate that in aLL cells, L-asparaginase-induced mPTP formation, which depends on IP3R-mediated ER Ca^2+^ release, involves the MCU channel that is linked to mitochondrial Ca^2+^ uptake (Giorgi et al., 2018).

**FIGURE 3:**
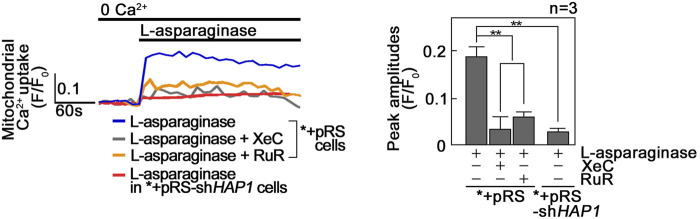
L-asparaginase causes Ca^2+^ transfer from the ER to the mitochondria. *+pRS and *+pRSsh*HAP1* cells loaded with Rhod-2 AM and pre-treated with XeC or RuR were stimulated with L-asparaginase and analyzed for mitochondrial Ca^2+^ uptake by spectrofluorometry. Data on the left is from one of three independent experiments showing similar results. Peak amplitudes were quantified as ratios of fluorescence (F/F_0_) after addition of L-asparaginase. F_0_ represents basal fluorescence or fluorescence before stimulation with L-asparaginase. The chart on the right shows mitochondrial Ca^2+^ uptake in *+pRS and *+pRS-sh*HAP1* cells treated as described above. Values are means ± SEM from the three independent experiments (*n* = 3). **p < 0.05.

We then examined if L-asparaginase induces a rise in mitochondrial Ca^2+^ level ([Ca^2+^]_mt_). To do so, *+pRS and *+pRS-sh*HAP1* cells loaded with the cell permeable mitochondrial Ca^2+^ dye, Rhod-2 AM^33^, were treated with L-asparaginase, and analyzed for mitochondrial Ca^2+^ increase by spectrofluorometry. As shown in Figure 3, L-asparaginase, which causes ER Ca^2+^ release in *+pRS cells (Figure 1C), induced mitochondrial Ca^2+^ increase in these cells, but not in HAP1-depleted ^*^+pRS-sh*HAP1* cells [where ER Ca^2+^ release is inhibited (Figure 1C)]. To further establish a link between L-asparaginase-induced IP3R-mediated ER Ca^2+^ release and increased [Ca^2+^]_mt_, *+pRS cells were pre-treated with Xestospongin C (XeC), a potent inhibitor of IP3R (Gafni et al., 1997), prior to L-asparaginase treatment, and spectrofluorometric Ca^2+^ analysis was performed. XeC dramatically reduced mitochondrial Ca^2+^ increase in *+pRS cells, indicating that the rise in [Ca^2+^]_mt_ is associated with L-asparaginase-induced ER Ca^2+^ release and subsequent transfer to the mitochondria. Pre-treatment with RuR also inhibited L-asparaginase-induced ER-mitochondria Ca^2+^ transfer, further indicating the involvement of MCU in the process.

**FIGURE 4:**
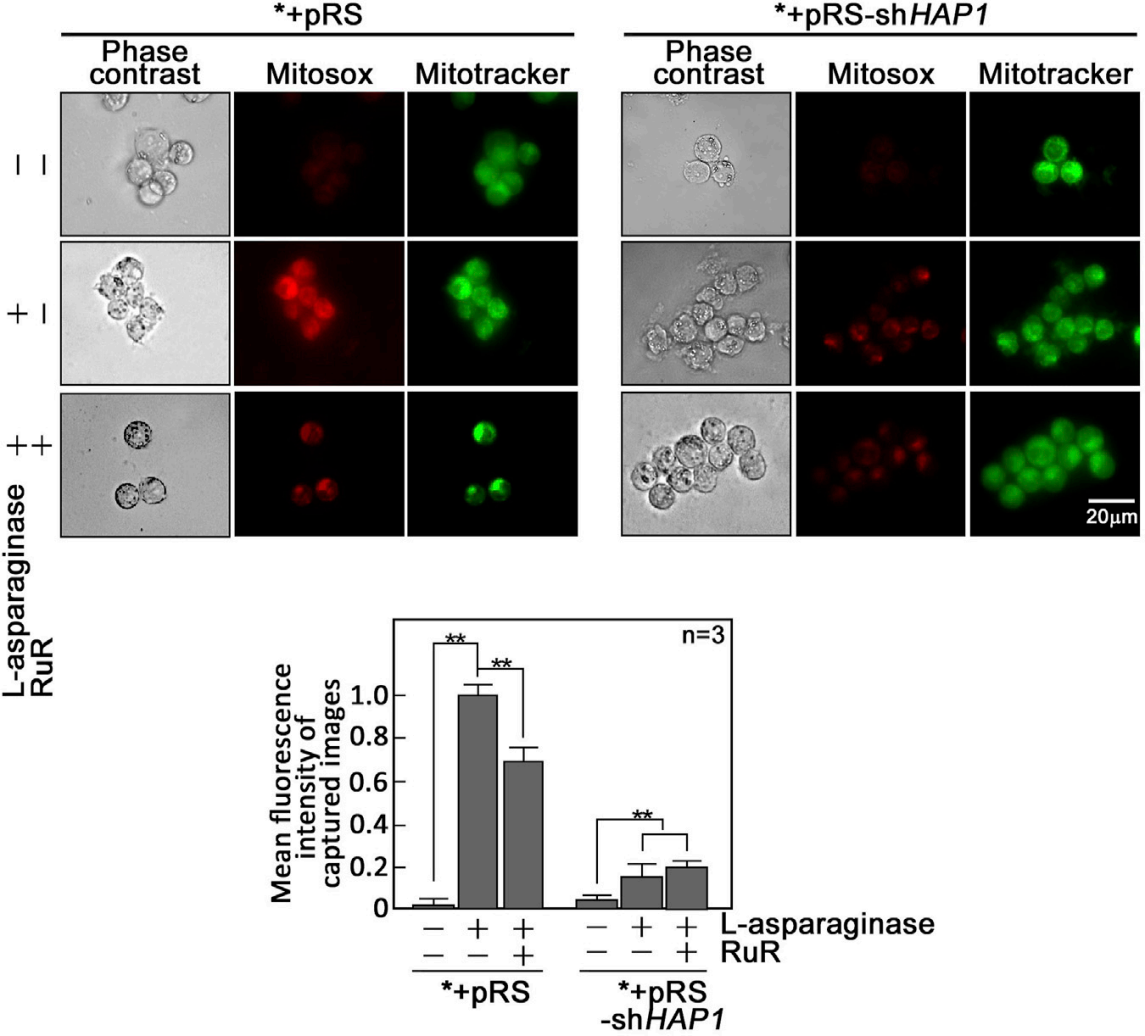
L-asparaginase induces an increase in mitochondrial ROS production. *+pRS and *+pRS-sh*HAP1* cells pre-treated with RuR then stimulated with L-asparaginase were stained with MitoSOX red and MitoTracker green, and examined by microscopy. Cell images were acquired using an Olympus 1 × 71 inverted microscope at ×360 magnification. Bar size = 20 µm. The bar graph shows mean fluorescence intensity of captured images measured using the ImageJ software with values from cells treated with L-asparaginase alone normalized to 1. Values are means ± SEM from three independent experiments (*n* = 3). **p < 0.05.

**FIGURE 5:**
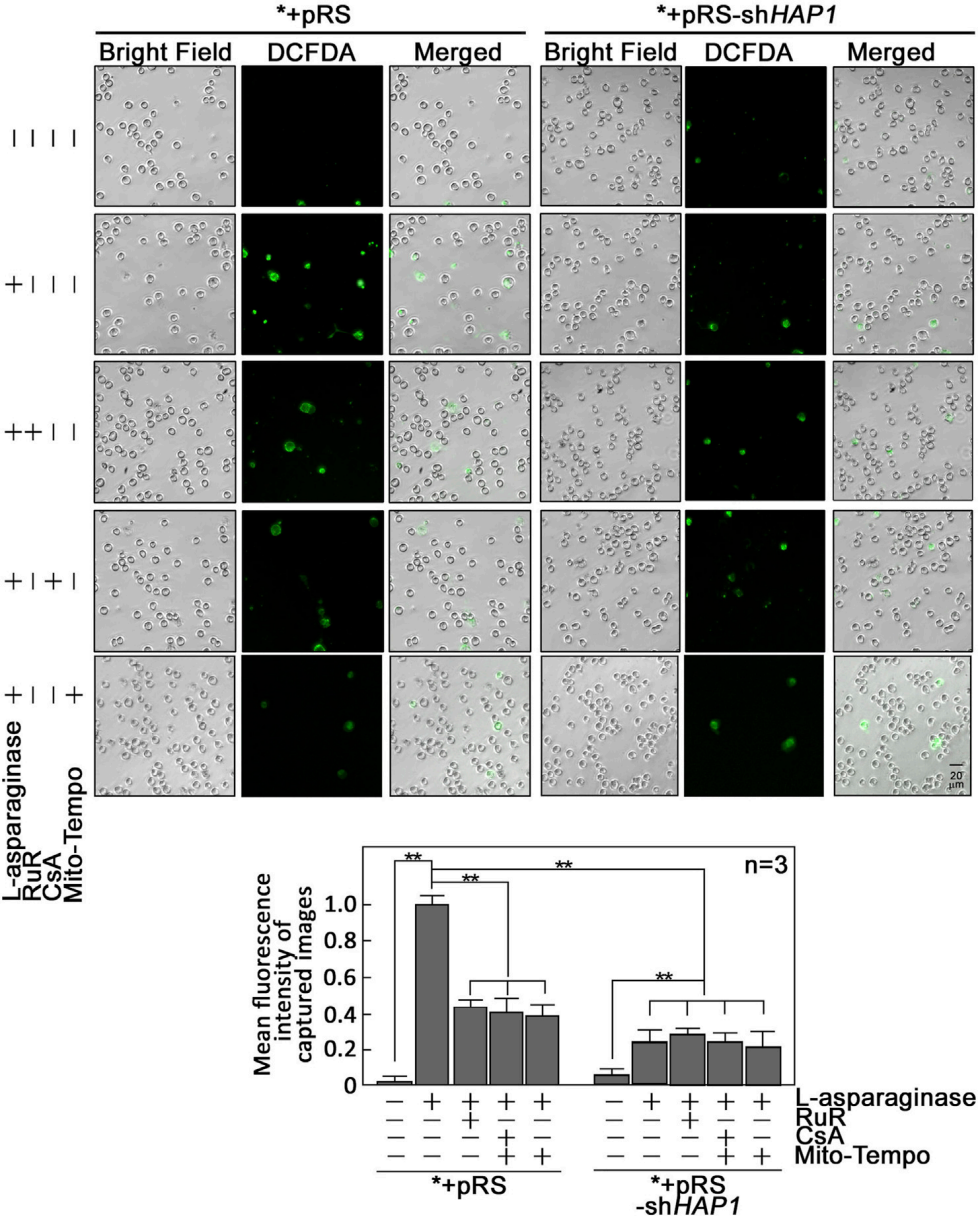
L-asparaginase causes an increase in cytoplasmic ROS level. *+pRS and *+pRS-sh*HAP1* cells pre-treated with XeC, RuR, CsA or Mito-Tempo then treated with L-asparaginase were stained with DCFDA and examined by microscopy. Cell images were acquired using an Olympus 1 × 71 inverted microscope at ×160 magnification. The bar graph shows mean fluorescence intensity of captured images measured using the ImageJ software with values from cells stimulated with L-asparaginase normalized to 1. Values are means ± SEM from three independent experiments (*n* = 3). **p < 0.05.

L-asparaginase-induced rise in [Ca^2+^]_mt_ evokes an increase in reactive oxygen species (ROS) in aLL cells. Since a rise in [Ca^2+^]_mt_ has been associated with the generation of mitochondrial ROS (Ermak and Davies, 2002; Gorlach et al., 2015), which also contributes to mPTP formation (Zorov et al., 2000; NavaneethaKrishnan et al., 2020) that allows ROS release into the cytoplasm (Zorov et al., 2014), we examined if L-asparaginase-induced rise in [Ca^2+^]_mt_ upregulates mitochondrial and cytosolic ROS levels in aLL cells. To determine mitochondrial superoxide anion levels, *+pRS and *+pRS-sh*HAP1* cells pre-treated with RuR, then stimulated with L-asparaginase were stained with MitoSOX and examined by microscopy. As shown in Figure 4, L-asparaginase induced a rise in mitochondrial ROS level in *+pRS cells, which was inhibited by pre-treatment with RuR and more so by HAP1 loss in *+pRS-sh*HAP1* cells. We then examined cytosolic ROS levels in *+pRS and *+pRS-sh*HAP1* cells pre-treated with RuR or CsA or Mito-Tempo, a mitochondrial ROS scavenger (NavaneethaKrishnan et al., 2018), then stimulated with L-asparaginase. Cells were stained with 2′,7′-dichlorofluorescin diacetate (DCFDA) and examined by microscopy. Figure 5 shows that L-asparaginase induced a rise in cytosolic ROS level in *+pRS cells, which was inhibited by pre-treatment with RuR, CsA or Mito-Tempo and more so by HAP1 loss in *+pRS-sh*HAP1* cells. These findings indicate that L-asparaginase-induced rise in [Ca^2+^]_mt_ is accompanied by mitochondrial and cytosolic ROS increases in aLL cells.

**FIGURE 6:**
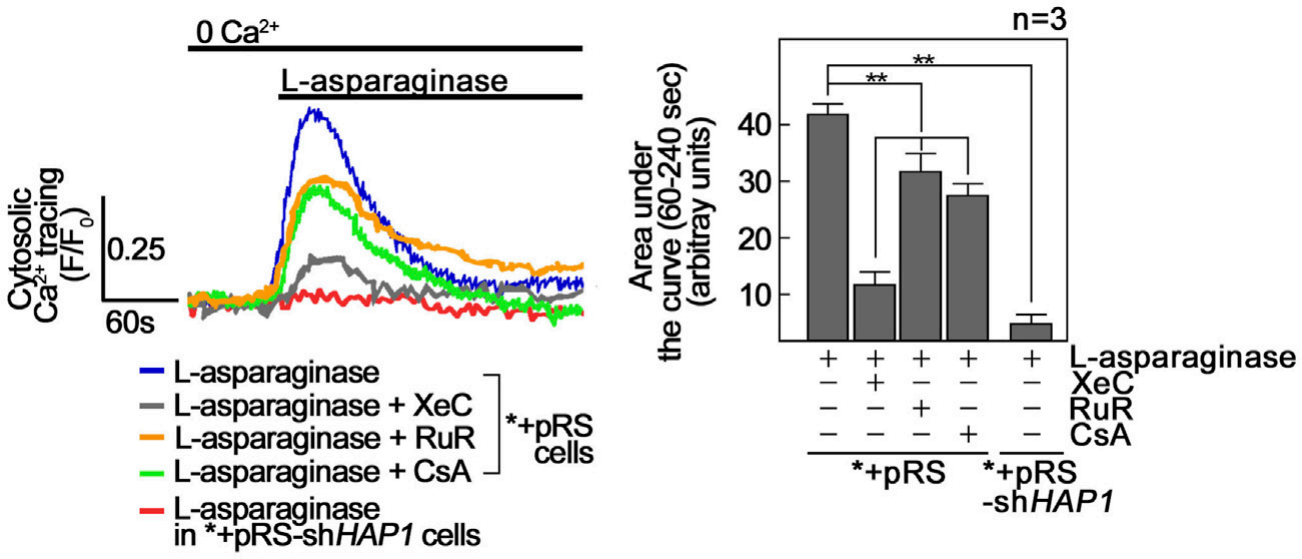
L-asparaginase causes a rise in [Ca^2+^]_cyt_. *+pRS and *+pRS-sh*HAP1* cells loaded with Fluo-4 AM and pre-treated with XeC, RuR or CsA then stimulated with L-asparaginase were analyzed by single-cell Ca^2+^ imaging. The left panel shows the average Ca^2+^ tracing from 10 cells measured every 2 s. Data are from one of three independent experiments showing similar results. The chart on the right shows integrated Ca^2+^ signals (area under the curve from 60 s to 240 s following treatment) in *+pRS and *+pRSsh*HAP1* cells treated as described above. Values are means ± SEM from the three independent experiments (*n* = 3). **p < 0.05.

L-asparaginase-induced ER-mitochondria Ca^2+^ transfer and subsequent mPTP formation cause a rise in [Ca^2+^]_cyt_. We then examined whether a rise in [Ca^2+^]_cyt_ observed in L-asparaginase-treated aLL cells (Lee et al., 2019) is due to ER-mitochondria Ca^2+^ transfer and subsequent mPTP formation. To do so, *+pRS cells loaded with Fluo-4 AM were pre-treated with XeC, RuR or CsA then treated with L-asparaginase and analyzed for Ca^2+^ transients by single-cell Ca^2+^ imaging. HAP1-depleted *+pRS-sh*HAP1* cells were again examined to test whether L-asparaginase-induced IP3R-mediated ER Ca^2+^ release is linked to a rise in [Ca^2+^]_cyt_. As shown in Figure 6, L-asparaginase caused an increase in [Ca^2+^]_cyt_ in *+pRS cells, which was inhibited by pre-treatment with RuR or CsA and more so by XeC, and by HAP1 loss in *+pRS-sh*HAP1* cells. These findings indicate that IP3R-stimulated ER Ca^2+^ release and transfer to the mitochondria, and subsequent mPTP formation account for L-asparaginase-induced rise in [Ca^2+^]_cyt_.

**FIGURE 7:**
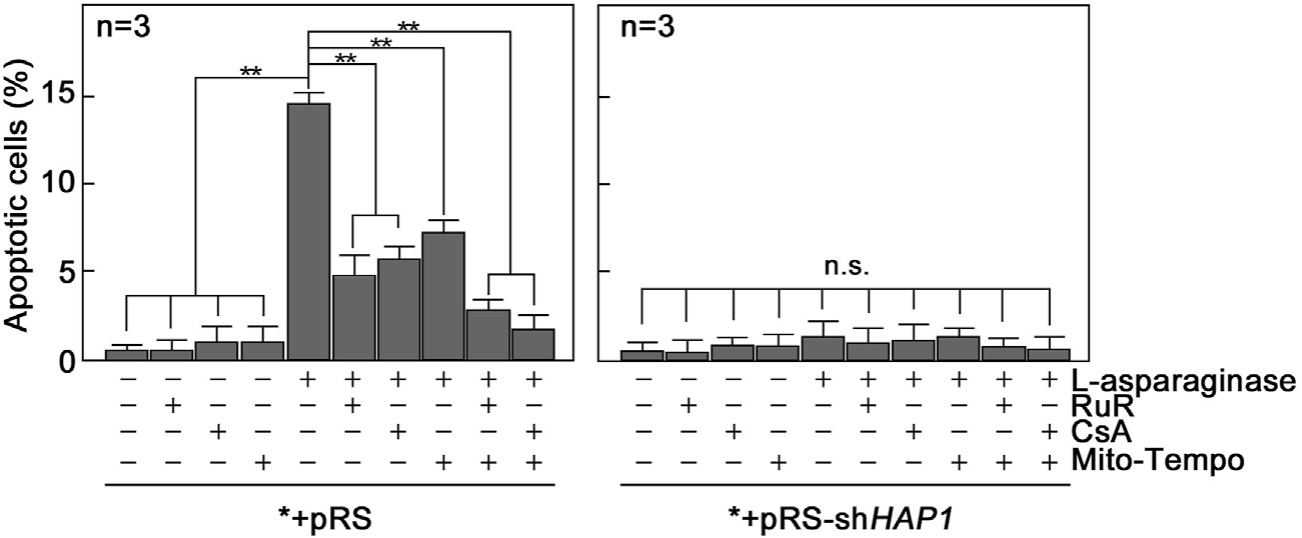
Blocking ER-mitochondria Ca^2+^ transfer by RuR, mPTP formation by CsA and/or mitochondrial ROS production by Mito-Tempo inhibit L-asparaginase-induced apoptosis. *+pRS and *+pRS-sh*HAP1* cells pre-treated with RuR, CsA or Mito-Tempo then stimulated with L-asparaginase were stained with Hoechst 34580 and FITC-Annexin V. FITC-positive apoptotic cells were counted 12 h post-treatment at ×10 magnification using a I×71 Olympus inverted microscope attached to a 37°C incubator with 5% CO_2_. The percentage of apoptotic cells was determined from a field of ∼100 Hoechst 34580-stained cells using the Olympus CellSens software (Olympus, Japan). Values are means ± SEM from three independent experiments (*n* = 3). **p < 0.05. N.S. not significant.

L-asparaginase-induced apoptosis is inhibited by blocking ER-mitochondria Ca^2+^ transfer, mPTP formation and/or mitochondrial ROS production. As indicated above, L-asparaginase causes aLL cell apoptosis by triggering IP3R-mediated ER Ca^2+^ release that results in a lethal rise in [Ca^2+^]_i_ and upregulation of the Ca^2+^-activated calpain-1-Bid-caspase-3/12 pathway (Lee et al., 2019). Treatment with the Ca^2+^ chelator, BAPTA-AM, in aLL cells reversed L-asparaginase-induced apoptotic cell death, indicating a link between [Ca^2+^]_cyt_ increase and apoptosis in aLL cells (Lee et al., 2019). Thus, we sought to determine whether L-asparaginase-induced ER-mitochondria Ca^2+^ transfer and subsequent ROS production, which cause mPTP opening that leads to increased [Ca^2+^]_cyt_, are linked to L-asparaginase-induced apoptosis in aLL cells. To do so, *+pRS cells pre-treated with RuR, CsA or Mito-Tempo then stimulated with L-asparaginase were stained with Hoechst 34580 and FITC-Annexin V. FITC-positive apoptotic cells were counted 12 h post-treatment and the percentage of apoptotic cells was determined. As shown in Figure 7, L-asparaginase-induced aLL cell apoptosis was inhibited by RuR, CsA or Mito-Tempo in *+pRS cells (left panel) but not in *+pRS-shHAP1 cells (right panel), which showed no ER Ca^2+^ release upon stimulation with L-asparaginase (Figure 1C). Altogether, our findings indicate that L-asparaginase-induced aLL cell apoptosis caused by an IP3R-mediated ER Ca^2+^ release and subsequent lethal rise in [Ca^2+^]_cyt_ (Lee et al., 2019) results from ER-mitochondria Ca^2+^ transfer and ROS production, which lead to mPTP formation.

## Discussion

Mitochondria regulate a number of cellular processes, including Ca^2+^ homeostasis and apoptosis. They communicate dynamically with the ER and store part of the released ER Ca^2+^. In this study, we demonstrate that L-asparaginase-induced IP3R-mediated ER Ca^2+^ release in aLL cells causes mPTP formation, which is inhibited in cells lacking HAP1 or upon inhibition of IP3R. These findings establish a link between L-asparaginase-induced IP3R-mediated ER Ca^2+^ release and mPTP formation. RuR inhibition of L-asparaginase-induced mPTP formation indicates the involvement of the MCU channel that is important for mitochondrial Ca^2+^ uptake (Giorgi et al., 2018). The fact that L-asparaginase also evokes [Ca^2+^]_mt_ increase in aLL cells, but not in those depleted of HAP1 or upon inhibition of IP3R suggests a link between L-asparaginase-induced Ca^2+^ transfer from the ER to the mitochondria and the rise in [Ca^2+^]_mt_.

Our finding that L-asparaginase-induced rise in [Ca^2+^]_mt_ is accompanied by mitochondrial and cytosolic ROS increase in aLL cells is consistent with the notion that a rise in [Ca^2+^]_mt_ contributes to mitochondrial ROS production (Ermak and Davies, 2002; Gorlach et al., 2015), which stimulates mPTP formation (Zorov et al., 2000; NavaneethaKrishnan et al., 2020) that facilitates the release of ROS into the cytoplasm (Zorov et al., 2014). Although we observed significant RuR inhibition of L-asparaginase-induced rise in both [Ca^2+^]_mt_ and mitochondrial ROS level in *+pRS cells, the degree of inhibition of mitochondrial ROS level is less than that of [Ca^2+^]_mt_. This difference may arise from the different time of measurement: Ca^2+^ response occurs within seconds and thus was measured immediately; on the other hand, ROS response is slower and was measured 12 h following L-asparaginase treatment. In addition, since ROS is produced in mitochondria and leaks into the cytoplasm through mPTP, there will be dynamic changes in mitochondrial levels of ROS which eventually accumulates in the cytoplasm. This explains the similar degree of RuR inhibition of [Ca^2+^]_mt_ and cytoplasmic ROS levels. Thus, differences in the extent of RuR inhibition in [Ca^2+^]_mt_ and mitochondrial ROS can be attributed to differences in method and time of measurement. Non-etheless, it is clear that MCU-mediated inhibition of [Ca^2+^]_mt_ increase also causes inhibition of ROS increase in both mitochondria and cytoplasm.

As for our view that the rise in [Ca^2+^]_cyt_ results from L-asparaginase-induced IP3R-mediated ER Ca^2+^ release and transfer to the mitochondria and subsequent mPTP formation, this is substantiated by the observed inhibition of the process in cells lacking HAP1 or when ER Ca^2+^ release, MCU, or mPTP is inhibited. Our finding that cells with inhibited MCU show greater [Ca^2+^]_cyt_ compared to cells with inhibited IP3R or depleted of HAP1 suggests that MCU inhibition causes released ER Ca^2+^ to bypass the mitochondria and go directly into the cytosol.

Overall, our findings align with previous studies showing that loss of cyclin-dependent kinase 5 (Cdk5) in breast cancer cells or knocking out Cdk5 in primary mouse embryonic fibroblasts (MEFs) is associated with mPTP formation, ROS increase [Ca^2+^]_mt_ and [Ca^2+^]_cyt_ increase, and caspase-mediated apoptosis (NavaneethaKrishnan et al., 2018; NavaneethaKrishnan et al., 2020). Our notion that L-asparaginase-induced apoptosis in aLL cells involves ER-mitochondria Ca^2+^ transfer, ROS production and mPTP formation is validated by inhibition of apoptosis upon inhibition of MCU channel, ROS production and/or mPTP formation.

**FIGURE 8:**
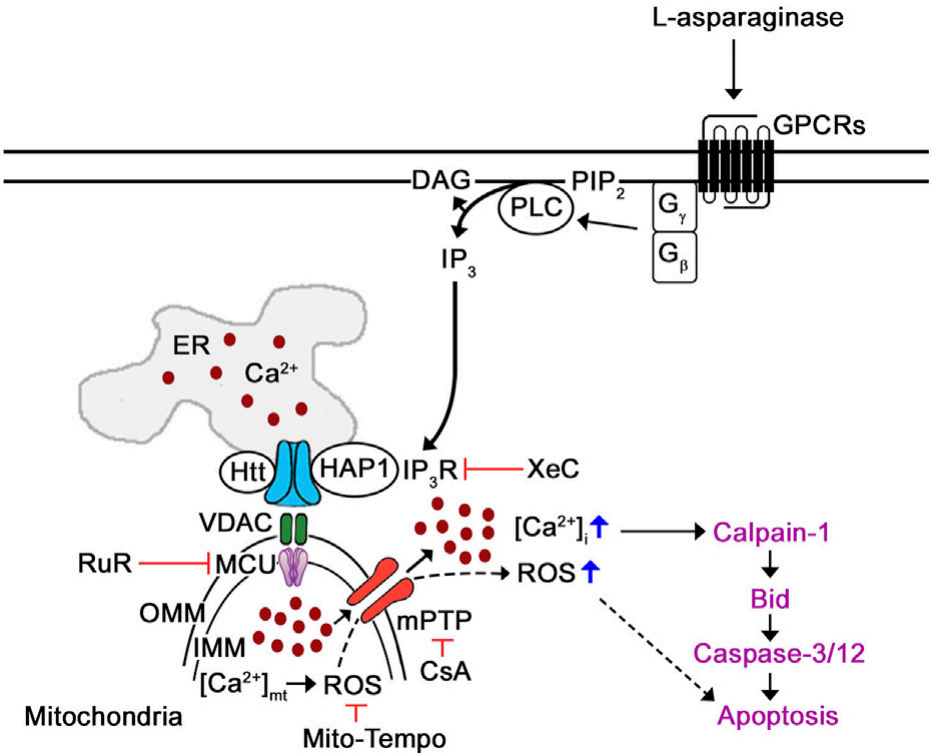
Proposed mechanism by which L-asparaginase induces mPTP-mediated aLL cell apoptosis *via* IP3R-dependent ER Ca^2+^ release. Previously, we have shown that L-asparaginase causes aLL cell apoptosis *via* the Ca^2+^-mediated calpain-1-Bid-caspase-3/12 apoptotic pathway (in purple) (Lee et al., 2019). In this study, we demonstrate that L-asparaginase-induced ER Ca^2+^ release triggers a rise in [Ca^2+^]_cyt_ by causing mitochondrial Ca^2+^ uptake and subsequent ROS production that leads to mPTP formation and subsequent aLL cell apoptosis. Since activation of opioid receptors has been shown to cause G_βγ_-mediated rise in [Ca^2+^]_i_ *via* PLC (Yoon et al., 1999; Charles et al., 2003; Celik et al., 2016; Machelska and Celik, 2018; Lee et al., 2021), we propose that L-asparaginase activation of GPCR (e.g., PAR2 (Peng et al., 2016)) in aLL cells causes G_βγ_-mediated stimulation of PLC, which triggers a rise in [Ca^2+^]_cyt_ through IP3R-mediated ER Ca^2+^ release, mitochondrial Ca^2+^ uptake and subsequent ROS production, causing mPTP opening that leads to aLL cell apoptosis.

Since stimulation of µ-opioid receptors (µ-ORs) was shown to trigger G_βγ_-mediated rise in [Ca^2+^]_cyt_ through phospholipase C (PLC) (Yoon et al., 1999; Charles et al., 2003; Celik et al., 2016; Machelska and Celik, 2018; Lee et al., 2021), we propose a model (Figure 8) whereby L-asparaginase causes aLL cell apoptosis *via* our previously identified Ca^2+^-mediated calpain-1-Bid-caspase-3/12 apoptotic pathway (in purple) (Lee et al., 2019). In this model, we show that L-asparaginase stimulates GPCR (e.g., PAR2 (Peng et al., 2016)) in aLL cells, causing G_βγ_-stimulation of PLC, which causes a rise in [Ca^2+^]_cyt_ through IP3R-mediated release of ER Ca^2+^ and mitochondrial Ca^2+^ uptake that leads to ROS production, which together induce mPTP formation.

In conclusion, our findings indicate that L-asparaginase-induced aLL cell apoptosis requires ER-mitochondria Ca^2+^ transfer, ROS production and mPTP formation. Thus, results from our studies not only fill in the gaps in our understanding of the Ca^2+^-mediated mechanisms by which L-asparaginase induces aLL cell apoptosis, but also offer a fresh perspective on targeting ER Ca^2+^ release, ER-mitochondria Ca^2+^ transport, ROS and/or mPTP in leukemic cells for aLL therapy.

## Data Availability

The original contributions presented in the study are included in the article/Supplementary Material, further inquiries can be directed to the corresponding author.
